# Vitamin E Attenuates the Progression of Non-Alcoholic Fatty Liver Disease Caused by Partial Hepatectomy in Mice

**DOI:** 10.1371/journal.pone.0143121

**Published:** 2015-11-24

**Authors:** Golnar Karimian, Marc Kirschbaum, Zwanida J. Veldhuis, Fernanda Bomfati, Robert J. Porte, Ton Lisman

**Affiliations:** 1 Surgical Research Laboratory, Department of Surgery, University of Groningen, University Medical Center Groningen, Groningen, The Netherlands; 2 Section of Hepatobiliary Surgery and Liver Transplantation, Department of Surgery, University of Groningen, University Medical Center Groningen, Groningen, The Netherlands; 3 Surgical Research Laboratory and Section of Hepatobiliary Surgery and Liver Transplantation, Department of Surgery, University of Groningen, University Medical Center Groningen, Groningen, The Netherlands; University of Basque Country, SPAIN

## Abstract

**Background and Aim:**

The progression of non-alcoholic fatty liver disease (NAFLD) likely involves a ‘multiple hit’ mechanism. We hypothesized that partial hepatectomy, a procedure performed frequently in patients with NAFLD, would accelerate the progression of disease.

**Methods:**

C57BL/6JolaHsd mice were fed a choline-deficient L-amino acid-defined diet (CD-AA) or a choline-sufficient L-amino acid-defined control diet (CS-AA). Part of the mice in the CD-AA group received a diet enriched in vitamin E (~20 mg /day). Two weeks after the start of the diet, mice underwent a partial hepatectomy or a sham operation.

**Results:**

In the CD-AA group, NAFLD activity scores were significantly higher at 7 days after partial hepatectomy compared to the sham operated mice (3.7 ± 1.3 vs. 1.8 ± 0.7; P<0.05). In addition, TBARS, a measure for oxidative stress, in liver tissue of the CD-AA group were significantly higher at day 1, 3 and 7 after partial hepatectomy compared to the sham operated mice (P<0.05). Vitamin E therapy significantly reduced TBARS level at day 7 after partial hepatectomy compared to the CD-AA diet group (P< 0.05). **V**itamin E suppletion reduced NAFLD activity score at day 7 after partial hepatectomy compared to the CD-AA group (2.3 ± 0.8 vs. 3.8 ± 1.0; P<0.05).

**Conclusion:**

Partial hepatectomy accelerates the progression of NAFLD. Disease progression induced by partial hepatectomy is substantially attenuated by vitamin E.

## Introduction

Non-alcoholic fatty liver disease (NAFLD) is one of the most common liver disorders worldwide [1]. NAFLD includes a disease spectrum ranging from simple steatosis to non-alcoholic steatohepatitis (NASH), which may eventually progress to liver fibrosis and cirrhosis [2]. Mechanisms of disease progression in NAFLD are incompletely understood, but likely a “multiple-hit” model is involved [2]. In this model, the development of steatosis increases the sensitivity of the liver to other hits such as oxidative stress and cytokines, leading to hepatocyte damage and liver dysfunction [3,4]. As the number of individuals with mild to moderate liver steatosis is increasing, it is reasonable to assume that the number of patients with steatosis that require a partial hepatectomy is increasing [5,6].

Partial hepatectomy is performed to manage many types of malignant or benign liver pathologies [7–9]. While a normal liver is capable to regenerate after a partial hepatectomy, severe steatosis and NASH impair liver regeneration and increase morbidity due to post-operative complications [5,10]. In contrast, several experimental and clinical studies indicate that mild steatosis does not impair liver regeneration [10–12]. Although the effect of steatosis on liver regeneration has been studied extensively, the effect of partial hepatectomy on the progression of NAFLD, to our knowledge, has not yet been investigated.

Liver regeneration after partial hepatectomy is associated with the activation of inflammatory signaling molecules and induction of oxidative stress [13,14]. Importantly, these factors are important contributors to the progression of NAFLD [3,15]. We hypothesized that partial hepatectomy would advance simple steatosis toward a progressive inflammatory phenotype and have investigated the effect of partial hepatectomy on the progression of the NAFLD in mice with mild steatosis. In the present study, we use a choline-deficient L-amino acid-defined (CD-AA) diet to induce mild steatosis in mice. We confirm previous studies that showed that mild steatosis does not impair liver regeneration. Importantly, we demonstrate that partial hepatectomy substantially accelerates the progression of NAFLD. In addition we show that anti-oxidant therapy with vitamin E attenuates the post-operative progression of NAFLD.

## Material and Methods

### Animal studies

C57BL/6JolaHsd male mice (5–6 weeks old, Harlan Laboratories, NL) were fed either a CD-AA diet (mouse chow 518753, Dyets Inc., PA, USA) or a CS-AA (mouse chow 518754, Dyets Inc., PA, USA). Animals were caged in animal rooms with an alternating 12-hour light/dark period, and had free access to food and water for the duration of the experiment. The local Committee for Care and Use of laboratory animals of the University of Groningen approved the experiments of this study and all the experiments were performed in accordance with the guidelines of this Committee.

### Experimental design

Two weeks after the start of the diet, mice were anesthetized using isoflurane/O_2_ and subjected to a midventral laparatomy with (PHx) or without (sham) a resection of two-thirds of the liver volume essentially as described previously [16]. Mice remained on the CD-AA or CS-AA diet after the partial hepatectomy or sham operation until the end of the experiment. Six mice in each group were sacrificed at selected time points. In an independent experiment, mice were fed the CD-AA diet for two weeks. At the end of the second week all mice were subjected to a sham or a PHx operation. After surgery, the mice were fed the CD-AA diet or the CD-AA diet enriched with vitamin E for one week (Vitamin E was provided in the form of DL-Alpha Tocopheryl Acetate with concentration of 5 mg vitamin E/gr food, mouse chow 519557, Dyets Inc., PA, USA). Prior to the experiment, we determined that the mice (20–25 gr) eat 4–5 grams of CD-AA diet each day. Thus, the mice received at least 20 mg vitamin E per day by having free access to food. We selected this dosage of vitamin E based on similar published experiments in rats with the goal to substantially improve the antioxidant status by feeding a high dosage of vitamin E to mice [17]. Nine mice in each group were sacrificed at day 7 after the operation.

At the time of sacrifice in all experiments, mice were weighed and anesthetized. Blood was harvested from the inferior *vena cava* after the injection of an anticoagulant solution containing 3.4% sodium citrate in 0.9% saline (8 μl/g) in the inferior *vena cava*. The remaining liver lobes were removed, weighed, and processed for biochemical or histological analyses.

### Liver histology and immunohistochemical staining

Paraffin-embedded liver sections (5-μm thick) were stained with standard hematoxylin and eosin (H&E) staining. These H&E sections were graded using the NAFLD scoring system [18]. At least 10 random high power fields (200X) were analyzed by two independent researchers. The NAFLD activity score was defined as an unweighted sum of scores for liver steatosis (<5 percent = 0, 5 to 33 percent = 1, >33 to 66 percent = 2, >66 percent = 3), lobular inflammation (no foci = 0, <2 foci per 200 X field = 1, 2 to 4 foci per 200 X field = 2, >4 foci per 200 X field = 3) and hepatocyte ballooning (none = 0, few balloon cells = 1, many cells/prominent ballooning = 2) [18]. Paraffin-embedded sections were also stained using Masson’s trichrome stain (Polysciences Inc., PA, USA) to assess presence of fibrosis. Hepatocyte proliferation was assessed by immunohistochemical staining of ki-67 on paraffin-embedded liver sections using a rabbit polyclonal antibody (abcam, Cambridge, UK). Proliferating hepatocytes were quantified by counting the number of nuclei positive for Ki-67, and were expressed relative to the total nuclei stained with hematoxylin per 200X field (hepatocyte proliferation index). At least six random high power fields were counted for each mouse.

### Biochemical studies in plasma and liver tissue

Plasma levels of alanine aminotransferase (ALT) and aspartate aminotransferase (AST) were assessed using standard biochemical methods. Thiobarbituric acid-reactive substances (TBARS) were measured in liver homogenates as a marker for oxidative stress. Samples were homogenized in cold 1.15% KCl buffer and TBARS were determined using malondialdehyde as a standard as previously described [19]. Protein concentration in liver homogenates was determined with a bicinchoninic acid (BCA) protein assay kit (Thermo Scientific, IL, USA) using bovine serum albumin as a standard.

### Real- time reverse transcriptase polymerase chain reaction

HO-1, CD68, MCP1 and NRF2 gene expressions in liver tissue of mice were determined by real-time reverse transcriptase polymerase chain reaction (RT-PCR) as was described previously [20]. Primer sets were designed using Primer Express 2.0 software (Applied Biosystems) and validated in a six step two-fold dilution series. For each gene the expression was normalized relative to the mean cycle threshold (CT) value of Beta-actin gene. Results were finally expressed as fold induction (2^[delta] CT^), an index of the relative amount of mRNA expression.

### Statistical analysis

GraphPad Prism (version 5.00) was used to perform all the analyses. Results are presented as the mean ± SD. A student t-test or a two-way analysis of variance (ANOVA) was used to determine the significance of differences between experimental groups. A P-value of less than 0.05 (P < 0.05) was considered statistically significant.

## Results

### The CD-AA diet induces mild steatosis in mice by 3 weeks

**Fig 1:**
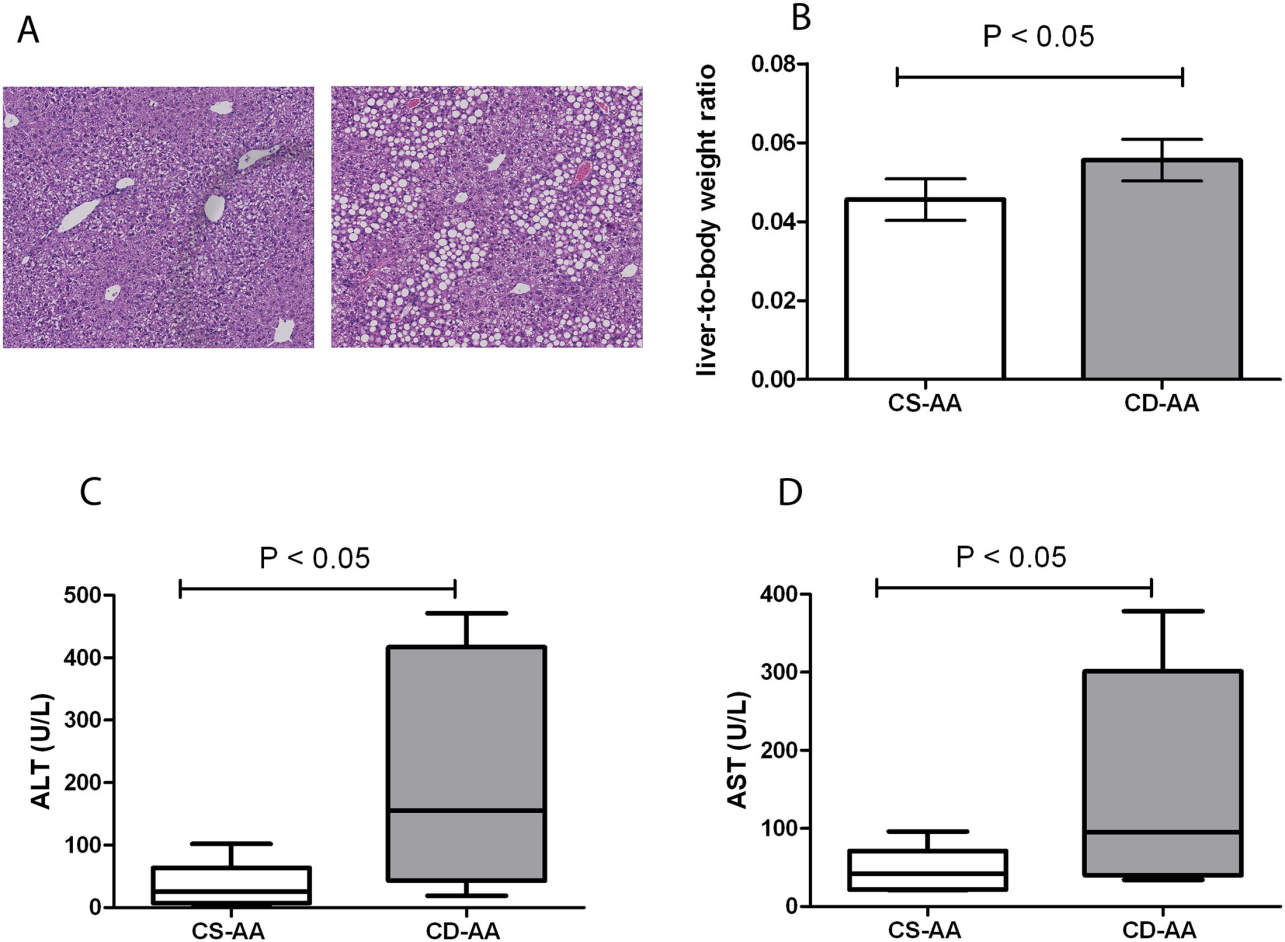
The choline-deficient L-amino acid-defined diet (CD-AA) induces mild steatosis by 3 weeks. (A) Hematoxylin and eosin staining of paraffin-embedded liver sections of mice on a CD-AA diet (right panel) and control diet (CS-AA, left panel) by 3 weeks (Magnification, X 100). (B) Liver-to-body weight ratios of mice on a CD-AA diet or a CS-AA diet by 3 weeks (n = 9). (C) Plasma levels of ALT and (D) AST in mice on a CD-AA diet or a CS-AA diet by 3 weeks (n = 9).

There are several models to induce liver steatosis in experimental animals. However, models such as a high fat diet and ob/ob mice show minimal steatohepatitis and liver fibrosis and therefore are not suitable models to study the progression of NAFLD [21]. However other diet models may develop NAFLD/NASH which is more similar to human disease. In order to investigate the effect of partial hepatectomy on the progression of NAFLD in mild steatotic livers, we used the CD-AA diet model. The CD-AA diet induces mild steatosis by 2–3 weeks in mice. Consequently, the NAFLD progresses over time and histopathological features of steatohepatitis such as lobular inflammation, hepatocyte ballooning and fibrosis appear in the liver [22]. In accordance with previous studies, the CD-AA diet induced mild steatosis by 3 weeks as was demonstrated by histological examination (Fig 1a) and an elevated NAFLD activity score (1.8 ± 0.7). The NAFLD activity score in the mice fed the CS-AA diet was 0 in all mice. In addition, the liver-to-body weight ratio was significantly higher in the CD-AA group compared to the CS-AA group (P <0.05, Fig 1b). Histological examination showed no liver fibrosis by 3 weeks in any of the mice (data not shown). Mice in the CD-AA group had significantly higher levels of ALT and AST in plasma compared to the CS-AA group by 3 weeks (Fig 1c and 1d, P <0.05). Altogether, our data indicate that the mice on the CD-AA diet had mild steatosis by 3 weeks.

### Mild steatosis does not impair liver regeneration after partial hepatectomy

**Fig 2:**
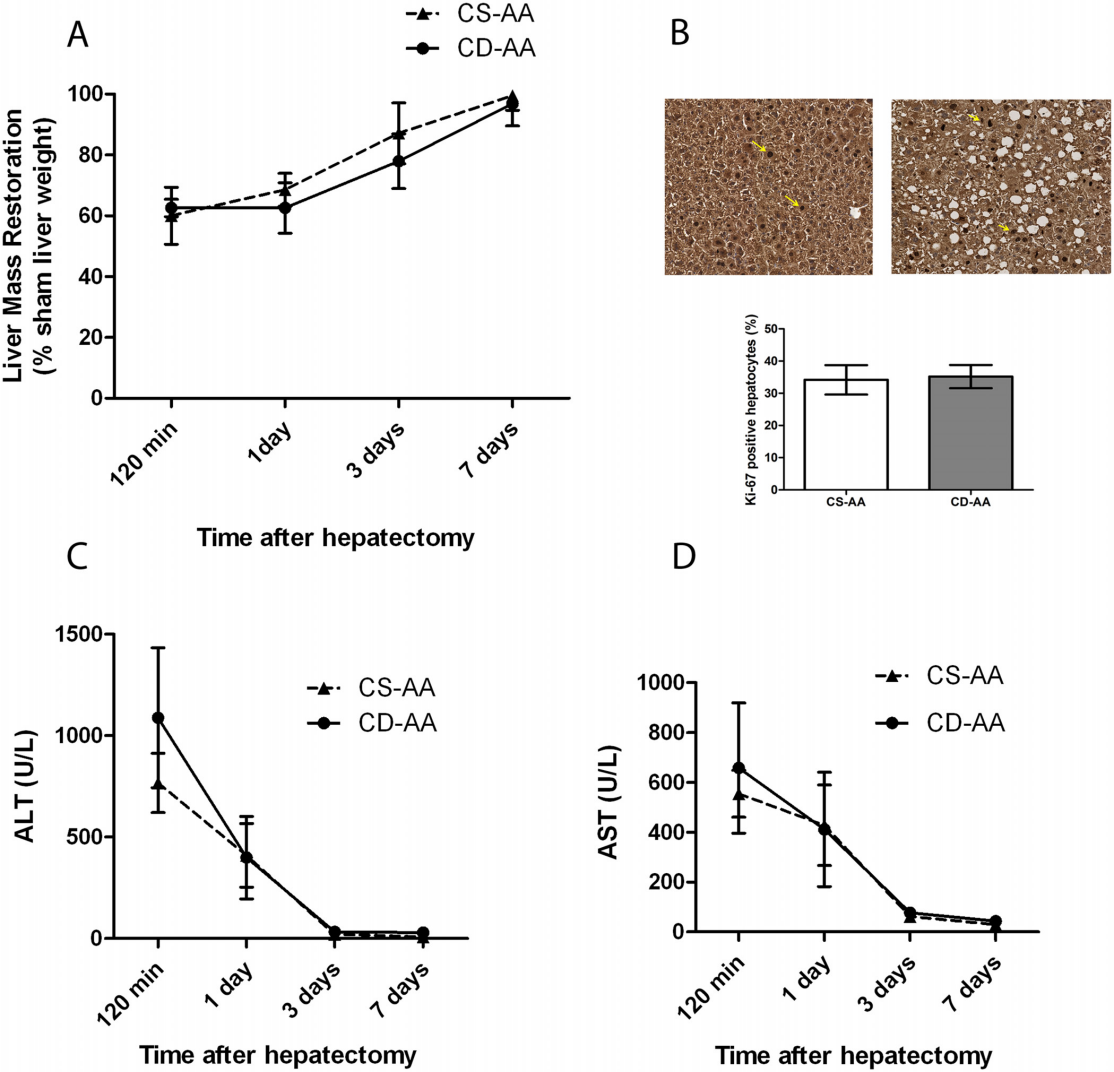
Mild steatosis does not impair liver regeneration after partial hepatectomy. (A) Graph represents the restoration of liver mass over the 7-days duration after partial hepatectomy. (B) Immunohistochemical staining of Ki-67 (yellow arrows show ki-67 positive cells) in mice on a CD-AA diet (right panel) or a CS-AA diet (left panel) at day 3 after hepatectomy (Magnification, X 200). Graph represents the number of nuclei positive for Ki-67 relative to the total nuclei stained with hematoxylin at day 3 after hepatectomy. At least six random high power fields were counted for each mouse. (C) Plasma levels of ALT and (D) AST in mice on a CD-AA diet or a CS-AA diet at different time points after hepatectomy (n = 6 per time point).

In order to investigate the effect of mild steatosis on the regenerative capacity of the liver, we performed a two-thirds partial hepatectomy on the mild steatotic livers of mice on the CD-AA diet or on the non-steatotic livers of mice on the CS-AA diet. We compared the liver mass restoration and hepatocyte proliferation rate at several time points after partial hepatectomy over a period of 7 days in both of the diet groups. Liver mass restoration over time was not different between the CD-AA and CS-AA groups (Fig 2a). The hepatocyte proliferation rate, as assessed by ki-67 staining, was similar in both steatotic and non-steatotic livers when assessed at day 3 after the hepatectomy (Fig 2b). In addition, aminotransferase levels in plasma increased significantly (P <0.05) after partial hepatectomy in both the CD-AA and CS-AA groups and returned to basal levels over time (Fig 2c and 2d). Aminotransferase levels were similar in the CD-AA and CS-AA group at all time points examined. Altogether, our data indicate that mild steatosis does not impair liver regeneration after partial hepatectomy.

### Partial hepatectomy accelerates the progression of NAFLD

**Fig 3:**
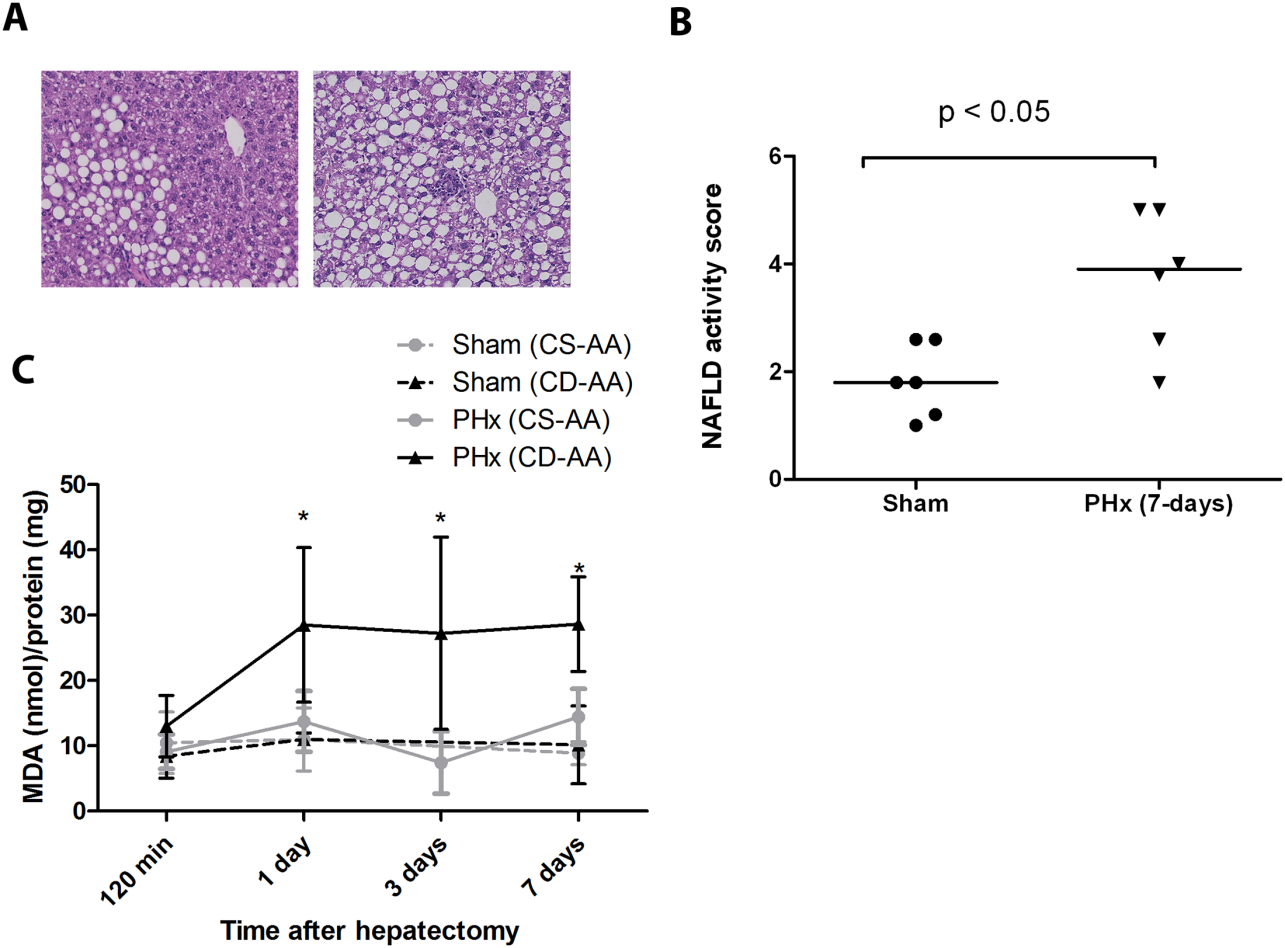
Partial hepatectomy accelerates the progression of NAFLD. (A) Hematoxylin and eosin staining of paraffin embedded-liver sections of mice on a CD-AA diet at day 7 after a partial hepatectomy (right panel) or a sham operation (left panel) (Magnification, X 200). (B) Graph represents the NAFLD activity scores in mice on a CD-AA diet at day 7 after a partial hepatectomy or a sham operation (n = 6 per group). (C) Graph represents the TBARS levels in the liver tissue at different time points after a partial hepatectomy (n = 6 per group) or a sham operation (n = 3 per group) in mice on CD-AA diet and CS-AA diet. Malondialdehyde (MDA) was used as standard. * P < 0.05 for CD-AA vs. CS-AA at day 1, day 3 and day 7 after partial hepatectomy, respectively.

To investigate the effect of a partial hepatectomy on the progression of NAFLD, we compared the histological scores of steatotic livers of mice on a CD-AA diet at 7 days after a partial hepatectomy or a sham operation (Fig 3a). In the CD-AA group, the NAFLD activity scores were significantly higher at 7 days after partial hepatectomy compared to the sham operated mice (3.7 ± 1.3 vs. 1.8 ± 0.7; P < 0.05; Fig 3b). Liver fibrosis was not present in both sham and hepatectomy groups at all the time points that were analyzed in our study (data not shown). The livers of mice on the CS-AA diet did not have steatosis, lobular inflammation, ballooning or fibrosis at 7 days after the hepatectomy or sham operation (data not shown). Altogether, our results indicate that partial hepatectomy accelerates the progression of NAFLD in mild steatotic livers.

### The level of lipid peroxidation increases significantly in steatotic livers after partial hepatectomy

Several experimental and human studies indicate that oxidative stress-mediated lipid peroxidation particularly contributes to the progression of NAFLD and acts as a “second-hit” [4,23,24]. Therefore, we measured TBARS to estimate the extent of lipid peroxidation in liver homogenates at several time points after partial hepatectomy. TBARS in liver tissue of the CD-AA group but not of the CS-AA group were significantly higher at day 1, 3 and 7 after partial hepatectomy compared to the sham mice (Fig 3c, P <0.05). These data suggest that oxidative stress level after partial hepatectomy increases particularly in steatotic livers.

### Vitamin E therapy after partial hepatectomy significantly reduces the oxidative stress level and attenuates the progression of NAFLD

**Fig 4:**
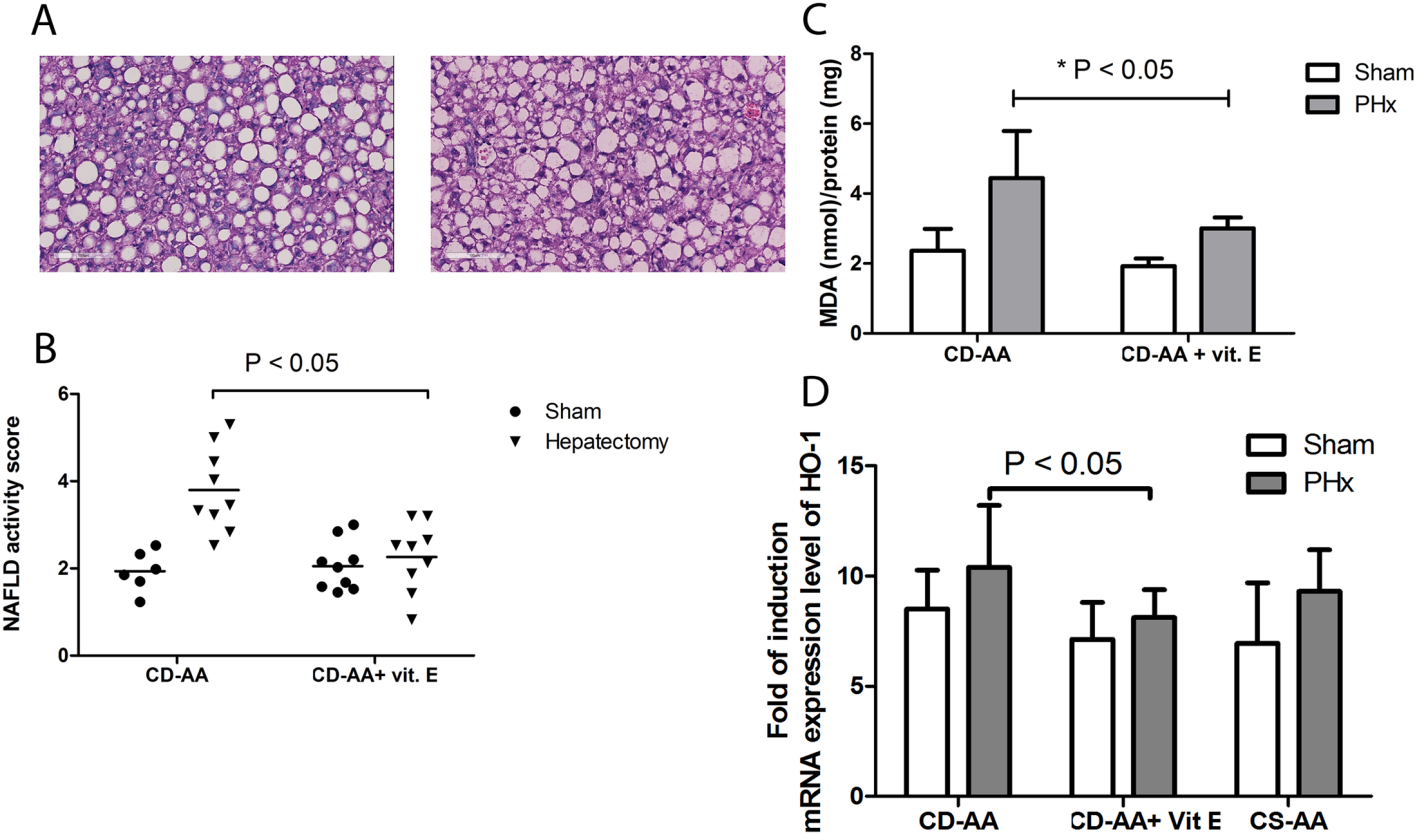
Vitamin E attenuates the progression of NAFLD after partial hepatectomy. (A) Hematoxylin and eosin staining of paraffin embedded-liver sections of mice on a CD-AA diet (right panel) or vitamin E enriched CD-AA diet (left panel) at day 7 after a partial hepatectomy (Magnification, X 200). (B) Graph represents the NAFLD activity scores in mice on a CD-AA diet or vitamin E enriched CD-AA diet at day 7 after a partial hepatectomy (n = 9 per group). (C) Graph represents the TBARS levels in the liver tissue at 7 days after a partial hepatectomy or a sham operation (n = 9 per group) in mice on CD-AA diet and vitamin E enriched CD-AA diet. Malondialdehyde (MDA) was used as standard. * P < 0.05 for CD-AA vs. vitamin E enriched CD-AA at day 7 after operation. (D) Graph represents the mRNA expression level of hemoxygenase-1 (HO-1) in the liver tissue at day 7 after a partial hepatectomy or a sham operation in mice on CS-AA diet (n = 6), CD-AA diet (n = 15) and vitamin E enriched CD-AA diet (n = 9). P< 0.05 for CD-AA vs Vitamin E enriched CD-AA at day 7 after operation.

A number of studies have suggested antioxidant therapy, particularly vitamin E, as an effective therapy against the progression of NAFLD [17,25,26]. Therefore, in an independent experiment, we treated the steatotic mice for one week after partial hepatectomy with high dosage of vitamin E to increase the antioxidant status of the liver. We compared the histological scores of steatotic livers of mice on a CD-AA diet and vitamin E enriched CD-AA diet at 7 days after a partial hepatectomy or a sham operation (Fig 4a). In this independent experiment, we confirm that partial hepatectomy accelerates disease progression in mice fed the CD-AA diet. In the vitamin E enriched CD-AA group, the NAFLD activity scores at 7 days after partial hepatectomy were significantly lower compared to the CD-AA diet group (2.3 ± 0.8 vs. 3.8 ± 1.0; P<0.05; Fig 4b). In addition, vitamin E therapy significantly reduced the TBARS level in liver tissue at day 7 after partial hepatectomy (P<0.05, Fig 4c) without delaying the liver regeneration (data not shown). We measured HO-1 (hemoxygenase -1) mRNA expression levels as a marker for oxidative stress at 7 days after the hepatectomy in CD-AA group and CD-AA Vitamin E group. We observed a significant decrease in HO-1 mRNA expression levels in Vitamin E treated group compared to the control group at day 7 after hepatectomy (Fig 4d). These data confirm our previously observed TBARS measurements.

We measured the triglyceride and cholesterol content in the liver tissue at day 7 after partial hepatectomy in CS-AA group, CD-AA group and vitamin E enriched CD-AA group. We observed a significant difference in the level of triglycerides and cholesterol between the CS-AA diet group and the other two groups. The triglyceride content at day seven after partial hepatectomy, however, was not significantly higher than sham operated mice (S1 Fig). Interestingly, the cholesterol content increased after the partial hepatectomy. These findings are in accordance with previous reports in the literature that partial hepatectomy does not affect the triglyceride content but cholesterol synthesis increases after the partial hepatectomy [27]. Vitamin E treatment did not affect the lipid content after partial hepatectomy. These results reinforce the notion that the increase in NAFLD activity score following partial hepatectomy and the inhibition of this effect by vitamin E does not reflect direct alterations of fat content of the liver, but rather reflects effects on intrahepatic inflammatory responses. We also measured the mRNA expression level of CD68 and MCP-1 in CD-AA group and Vitamin E enriched CD-AA group at day 7 after partial hepatectomy. We did not observe any significant difference in the expression levels of these markers at these time points (S1 Fig)

Altogether, in accordance with previous studies [17,25,26], our data suggests that vitamin E acts as an antioxidant and attenuates the progression of NAFLD after partial hepatectomy.

## Discussion

In this study, we report that mild liver steatosis does not impair liver regeneration in mice. However, partial hepatectomy substantially accelerates the progression of NAFLD. In addition, we provide evidence that enhanced oxidative stress following partial hepatectomy contributes to the progression of NAFLD and show that antioxidant therapy with vitamin E attenuates the progression of NAFLD.

The clinical significance of liver steatosis on liver regeneration after hepatic resection depends upon the extent of lipid accumulation in hepatocytes and the presence of steatohepatitis [5,6]. Several human studies have indicated that mild steatosis does not impair liver regeneration [10,11]. However, in experimental models it is difficult to distinguish between the effects of simple steatosis and the effects of a progressive phenotype of NAFLD on liver regeneration since different models show different histopathological features of NAFLD, and rodents have high tolerance before they display inflammatory phenotypes even in a severe steatotic status [21]. Therefore, data emerging from experimental studies on the effect of steatosis and steatohepatitis on regeneration are inconsistent [12,28]. In our study, we used the CD-AA diet model, which is suggested to be a model for simple steatosis with the ability to advance toward steatohepatitis over time, mimicking the natural history of NAFLD/NASH in humans [22]. In accordance with previous studies [10–12], we found that mild steatosis does not impair liver regeneration after partial hepatectomy.

Nevertheless, the development of liver steatosis sensitizes the liver to other hits [2]. In our study, we found that a mild steatotic phenotype could advance to a progressive inflammatory phenotype of NAFLD after partial hepatectomy. In addition, the level of lipid peroxidation products following partial hepatectomy was particularly higher in steatotic livers. Many experimental and human studies have reported a positive association between increased levels of the oxidative stress-mediated lipid peroxidation and the presence of progressive inflammatory phenotypes of NAFLD [3,23,29]. Several mechanisms have been suggested for the role of oxidative stress in the pathogenesis of NASH. Peroxidation of the mitochondrial/plasma membrane directly leads to necrosis/apoptosis of liver cells [30]. ROS-induced Fas-ligand expression on hepatocytes may lead to hepatocyte apoptosis given that isolated hepatocytes from liver biopsies of NASH patients express higher levels of Fas [31]. Covalent binding of the aldehyde products of lipid peroxidation (such as MDA) with hepatic proteins may lead to injurious immune responses in the liver [3]. Activation of the NF-κB cascade by products of lipid peroxidation can increase transcription of inflammatory cytokines and death ligands by liver cells [32]. Although we have not studied these mechanisms, they potentially explain why an increased level of lipid peroxidation after partial hepatectomy particularly contributes to the progression of NAFLD following a partial hepatectomy.

These findings are important for clinical practice because they implicate that simple hepatic steatosis, which is a benign condition and non-progressive in the majority of patients, may advance toward a more progressive liver disease after hepatic resection. This may have adverse clinical consequences for humans with simple steatosis that may need a partial hepatectomy and studies to examine whether acceleration of disease progression also occurs in humans with appreciable steatosis who undergo a partial hepatectomy are therefor warranted. Currently, few therapies are available to prevent the progression of NAFLD [1]. The prominent role of oxidative stress in NAFLD pathogenesis has prompted the use of antioxidants, particularly vitamin E, as an effective therapy in patients with progressive phenotypes of NAFLD. It has been reported that vitamin E improves all the histological features of NAFLD (except for fibrosis) and decreases the NAFLD activity score when compared with placebo [25,26]. We also showed in the present study, that vitamin E is a beneficial therapy to reduce the level of oxidative stress after partial hepatectomy and to prevent the progression of NAFLD. Would our findings be confirmed in humans, it may be of interest to examine whether patients with simple steatosis benefit from interventions such as vitamin E administration after partial hepatectomy.

In summary, our findings indicated that despite the intact regenerative capacity of mild steatotic livers, these livers are predisposed to advance to a more progressive inflammatory phenotype of NAFLD after partial hepatectomy. In addition, oxidative stress plays a pivotal role in disease progression and vitamin E may be a beneficial therapy in preventing the advance of steatosis to steatohepatitis after hepatic resection.

## Supporting Information

S1 Fig Triglycerde (A) and total cholesterol (B) in liver at day 7 after partial hepatectomy in CS-AA group, CD-AA group and CD-AA+ vitamin E group. Panels C,D,E represent the mRNA expression levels of CD-68, MCP-1 and nrf2 in liver at day 7 after partial hepatectomy.
(TIF)

S1 Text CD-AA and CS-AA diet composition used in the study. (PDF)

S2 Text Vitamin E enriched CD-AA diet composition used in the study. (PDF)

## Abbreviations

NAFLD: nonalcoholic fatty liver disease
NASH: nonalcoholic steatohepatitis
CD-AA: choline-deficient L-amino acid-defined diet
CS-AA: choline-sufficient L-amino acid-defined diet
TBARS: thiobarbituric acid-reactive substances
PHx: partial hepatectomy
H&E: hematoxylin and eosin staining
ALT: alanine aminotransferase
AST: aspartate aminotransferase
