# Supplementary figures and images for: Vitamin E Attenuates the Progression of Non-Alcoholic Fatty Liver Disease Caused by Partial Hepatectomy in Mice
Source: PLoS One. 2015 Nov 24;10(11):e0143121. doi: 10.1371/journal.pone.0143121 (PMC4658046; doi:10.1371/journal.pone.0143121)

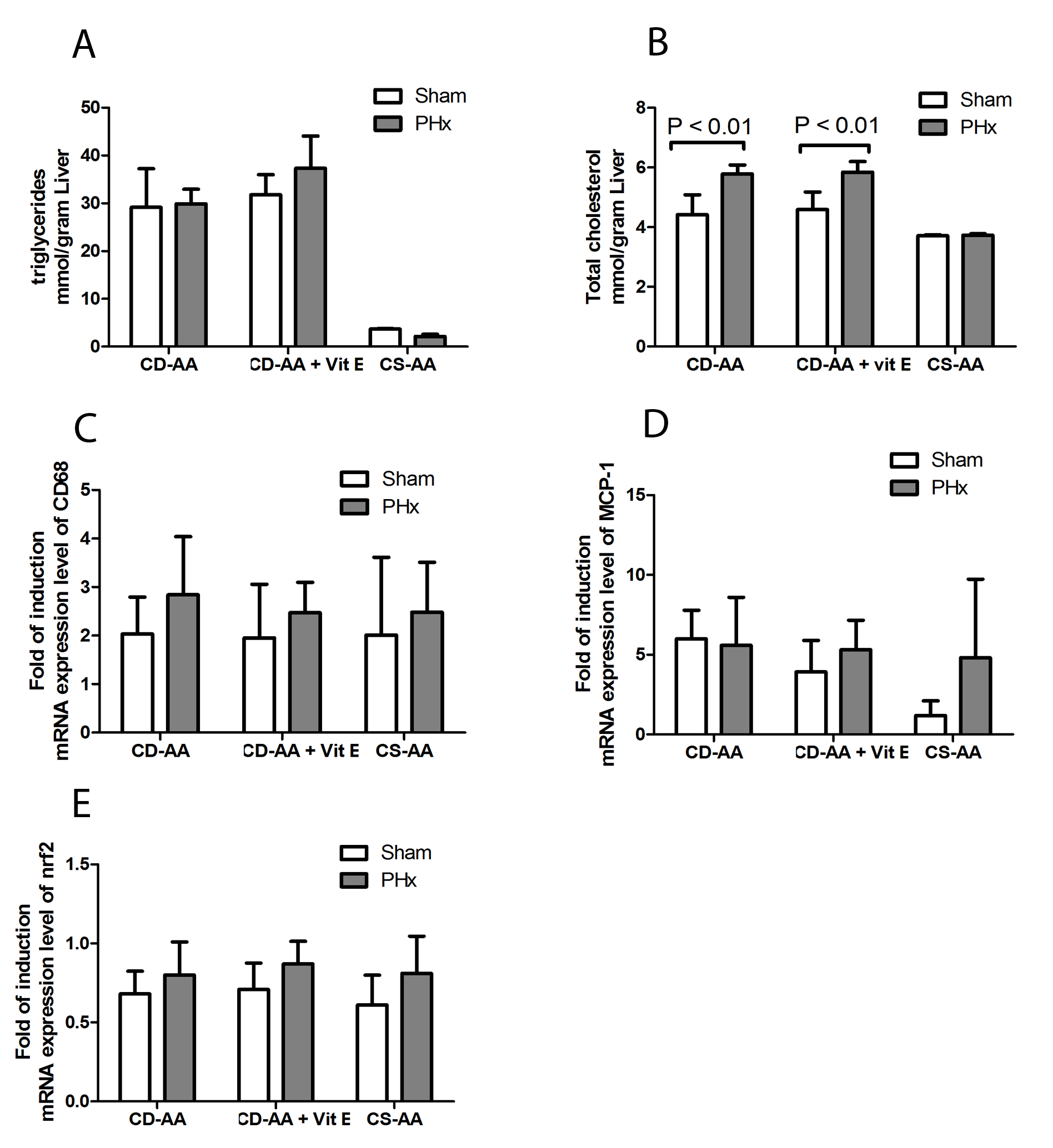

Supplement: S1 Fig — Triglycerde (A) and total cholesterol (B) in liver at day 7 after partial hepatectomy in CS-AA group, CD-AA group and CD-AA+ vitamin E group. Panels C,D,E represent the mRNA expression levels of CD-68, MCP-1 and nrf2 in liver at day 7 after partial hepatectomy. (TIF) [file pone.0143121.s001.tif]
