# Supplementary material for: Vitamin E Attenuates the Progression of Non-Alcoholic Fatty Liver Disease Caused by Partial Hepatectomy in Mice
Source: PLoS One. 2015 Nov 24;10(11):e0143121. doi: 10.1371/journal.pone.0143121 (PMC4658046; doi:10.1371/journal.pone.0143121)
Supplement: S1 Text — (PDF) [file pone.0143121.s002.pdf]

Dyets Inc.

2508 Easton / Bethlehem, Pennsylvania 18017
Within 610 Area Code: 868-7701, FAX -868-5170
Outside 610 Area Code: 800-275-3938
FAX: 800-329-3938
E-Mail Address: dyets@enter.net

Diet # 518753 & 518754

Choline Deficient and Sufficient Iron Supplemented L-AA Defined Rat Diet

			**518753**<br>**518754**	
L-Alanine			5.1	
L-Arginine			12.7	
L-Aspartic Acid			15.8	
L-Cystine			3.7	
L-Glutamic Acid			28.9	
Glycine			6.2	
L-Histidine			3.4	
L-Isoleucine			6.1	
L-Leucine			10.5	
L-Lysine-HCl			9.1	
L-Methionine			1.7	
L-Phenylalanine			7.3	
L-Proline			7.6	
L-Serine			7.2	
L-Threonine			4.6	
L-Tryptophan			1.8	
L-Tyrosine			5.7	
L-Valine			6.3	
**kcal/gm**	**4**	**total L-AA*……….**	**143.7**	**574.8**

			**518753**		**518754**	
**Ingredient**	kcal/gm		**gm/Kg**	**kcal/gm**	**gm/Kg**	**kcal/gm**
Cornstarch	3.6		100	360	100	360
Dextrin	3.63		100	363	100	363
Sucrose	4		406.67	1626.68	392.19	1568.76
Cellulose, Microcrystalline	0		50	0	50	0
Corn Oil	9		50	450	50	450
Primex	9		100	900	100	900
Salt Mix #215001 (no Fe Added)	0.47		35	16.45	35	16.45
Sodium Bicarbonate	0		4.3	0	4.3	0
Vitamin Mix #300050	3.87		10	38.7	10	38.7
Choline Bitartrate	0		0	0	14.48	0
Ferric Citrate, U.S.P.	0		0.33	0	0.33	0
		other total	856.30	3754.83	856.30	3696.91
		**grand total**	**1000.00**	**4329.63**	**1000.00**	**4271.71**

Dr. D. Nakae, Nara Medical College, May 10, 1989
